# Efficacy and safety of a multifactor intervention to improve therapeutic adherence in patients with chronic obstructive pulmonary disease (COPD): protocol for the ICEPOC study

**DOI:** 10.1186/1745-6215-12-40

**Published:** 2011-02-14

**Authors:** Pilar Barnestein-Fonseca, José Leiva-Fernández, Francisca Vidal-España, Antonio García-Ruiz, Daniel Prados-Torres, Francisca Leiva-Fernández

**Affiliations:** 1 Family and Community Medicine Teaching Unit of Malaga, Distrito Sanitario Málaga, Málaga, Spain; 2 Vélez Sur Health Centre, Área Sanitaria Málaga Este-Axarquía, Vélez Málaga (Málaga), Spain; 3 Farmacoeconomy and Clinical Therapeutic Department, Faculty of Medicine, Malaga University, Málaga, Spain

## Abstract

**Background:**

Low therapeutic adherence to medication is very common. Clinical effectiveness is related to dose rate and route of administration and so poor therapeutic adherence can reduce the clinical benefit of treatment. The therapeutic adherence of patients with chronic obstructive pulmonary disease (COPD) is extremely poor according to most studies. The research about COPD adherence has mainly focussed on quantifying its effect, and few studies have researched factors that affect non-adherence. Our study will evaluate the effectiveness of a multifactor intervention to improve the therapeutic adherence of COPD patients.

**Methods/Design:**

A randomized controlled clinical trial with 140 COPD diagnosed patients selected by a non-probabilistic method of sampling. Subjects will be randomly allocated into two groups, using the block randomization technique. Every patient in each group will be visited four times during the year of the study. Intervention: Motivational aspects related to adherence (beliefs and behaviour): group and individual interviews; cognitive aspects: information about illness; skills: inhaled technique training. Reinforcement of the cognitive-emotional aspects and inhaled technique training will be carried out in all visits of the intervention group.

**Discussion:**

Adherence to a prescribed treatment involves a behavioural change. Cognitive, emotional and motivational aspects influence this change and so we consider the best intervention procedure to improve adherence would be a cognitive and emotional strategy which could be applied in daily clinical practice. Our hypothesis is that the application of a multifactor intervention (COPD information, dose reminders and reinforcing audiovisual material, motivational aspects and inhalation technique training) to COPD patients taking inhaled treatment will give a 25% increase in the number of patients showing therapeutic adherence in this group compared to the control group.

We will evaluate the effectiveness of this multifactor intervention on patient adherence to inhaled drugs considering that it will be right and feasible to the clinical practice context.

**Trial registration:**

Current Controlled Trials ISCTN18841601

## Background

As with all chronic diseases, non-adherence in patients with COPD is common and contributes to adverse health outcomes, reduced quality of life and increased healthcare expenditures [1]. According to the World Health Organization (WHO), patient adherence to long term therapy averages 50 percent [2]. Adherence rates in clinical trials may be as high as 70 to 90 percent [3-5], but in clinical practice it is in the range of 10 to 40 percent [6-8]. In the Lung Health Study [3,9], therapeutic adherence with inhaled treatment recorded by self-reported methods after a one year follow-up was 60 percent and decreased to 50 percent at the five year follow-up.

Despite the research about treatment adherence, few factors have been associated with it [1]. Characteristics of COPD medications and regimens can contribute to non-adherence; several studies have reported that an average of 60 percent of patients with COPD do not follow the prescribed therapy [10]. Patients with COPD often have medication for other pathologies and polypharmacy is a common contributor to poor adherence. Another factor is the frequency of dosing for every drug, the more doses they have, the lower the adherence can be. Patients taking multiple medications, each one with a different dosing pattern, can be confused and frustrated by this and it can lead to forgetting to take medications. The route of administration can also influence adherence. The oral forms are easier and have higher adherence than inhaled medications [11]. In addition, mistakes in inhalation techniques are frequent and they too affect adherence; several studies have reported that up to 85 percent of patients use their inhaler ineffectively [10]. Another problem is the drug's efficacy; adherence is higher to a drug whose pharmacological effect is quick and has a direct impact on symptoms. Additional factors can include adverse side effects and, in some healthcare systems, the inability to pay or a reduced access to medication [12].

There are factors related with patients that can influence adherence such as age. Adherence is often better in older persons, but the increase of age is also associated with comorbidity, polypharmacy, cognitive decline and in some cases, with difficulty in reading small print or to open and manage the devices. Another factor is the patient's disease perception that leads to the discontinuation of therapy due to a lack of or excess of clinical symptoms, inconvenient or expensive medication [12]. This perception is built up by the patient and also by the healthcare provider and caregivers.

There are few studies about interventions to improve therapeutic adherence in COPD patients and other important aspects of the disease management. Most of them have been joint studies with asthmatic patients [13-18]. They have mainly focussed on quantifying the adherence and not many have explored factors which affect non-adherence, although the authors point out the need for working in motivational and cognitive ways to improve patients' understanding of both their illness and treatment [18].

Specific studies which consider educational programs exclusively for COPD patients, show significant improvement in patients' management of disease, less exacerbations, decrease in the rescue medication and improved knowledge about inhaler use [15,17]. These programs include information about the illness, the treatment, the importance of the adherence, the management of exacerbation, instructions about the inhalation technique, and recommendations about the proper time to ask for doctor's advice and, in some cases, counseling to cease smoking or referrals to independent programs.

Between 1998 and 2003 our research team carried out a cohort study of patients with COPD in two health centers in Malaga. The Batalla Test [19], the Morinsky-Green test [20] and the self-reported compliance from Haynes and Sackett test [21] were all included as treatment adherence evaluation methods. We found little consistent data. Between 2004 and 2006, we performed another cohort study in 195 COPD patients from 4 health centers. Patients have been followed up during 1 year and we have used four therapeutic adherence measures: the Batalla test, the Morinsky-Green test, the Haynes-Sackett test and the dose/pill count. In this study we selected the dose/pill count as the reference method to measure adherence and we calculated the diagnostic validity of the self-reported methods. We found that the use of the Batalla test and the Morisky-Green test together was the most reliable way to assess adherence by self-reported methods and we proposed a strategy to measure adherence in clinical practice depending on the adherence prevalence [22]. Taking all this into account, our next step in the study of adherence was the design of a multifactor intervention to improve treatment adherence in patients with COPD.

The main objective of this study is to evaluate the effectiveness of a multifactor intervention (COPD information, dose reminders, audiovisual material, motivational aspects and inhalation techniques training) to improve the therapeutic adherence in COPD patients with inhaled scheduled treatment after one year follow-up with two reinforcement visits (3 and 6 month after intervention). The second objective is to describe the main factors related to adherence. The specific aims are:

- To evaluate in two study groups treatment adherence prevalence with inhaled medication (primary outcome variable) at the beginning of the study, at three and six months and at one year after the intervention.

- To evaluate the functional status using a forced spirometry and the quality of life (St George Respiratory questionnarie, EuroQoL-5D) and the clinical progress (SeguiEPOC) as secondary outcome measures in both groups.

- To analyse the main factors related to therapeutic adherence.

## Methods

This study has been approved by the Ethical Committees of Distrito Sanitario Málaga (01/03/07) and Axarquía (13/05/08) and by Committee of Clinical Trials of Hospital Clínico Universitario Virgen de la Victoria (30/11/07). The study protocol was reviewed by Spanish Medicine Agency and Sanitary products.

### Participants

140 patients with COPD selected by a non-probabilistic consecutive sampling method. The inclusion criteria were searched in the patient's clinical record as: having been diagnosed of COPD by spirometry following the SEPAR (Sociedad Española de Neumología y Cirugia Torácica) guidelines [23], receiving clinical assistance in primary care centers in Malaga area, having been prescribed inhaled and scheduled treatment; and having agreed to be part of the study by giving signed written consent. Exclusion criteria: other respiratory conditions which are not included in the COPD definition (bronchiectasis, asthma or cystic fibrosis) and cognitive impairment problems registered in their clinical record (Dementia, Alzheimer, Parkinson, Cognitive decline).

### Sample size

it was calculated to detect an adherence percentage difference between the two groups of 25%, with a statistical power of 80% and a confidence level of 95%, assuming a percentage of expected losses of 15%. The final sample size was 140 patients with COPD that meet the selection criteria mentioned above.

### Design

**Figure 1:**
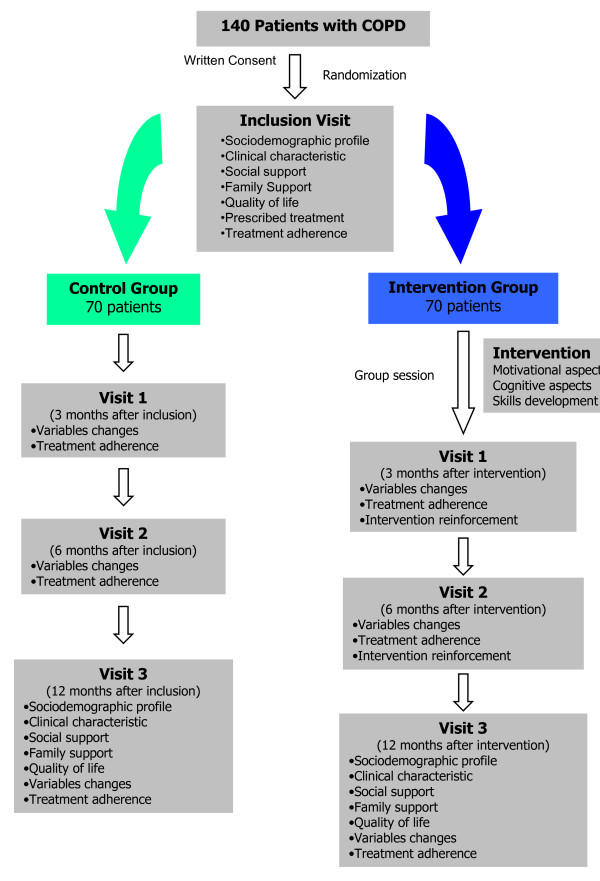
**Study general graphic**.

Randomized controlled clinical trial. (Figure 1)

### Setting

Primary Care

### Outcomes

#### Primary outcome

the therapeutic adherence considered as the percentage of patients classified as good adherent. The adherence will be measure by three adherence measurements: the Morinsky-Green test (MGT) [20], the Batalla test (BT) [19] and the dose/pills count. We will consider as reference methods the results of dose/pill count. Each method will be considered independently.

The MGT measures the attitude towards treatment and it was adapted by us to inhaled medication. The test has four questions and a patient will be considered a good adherent when he answers properly the complete questionnaire.

The BT provides information about the patients' knowledge of their illness. The test has three questions, adapted to COPD, and a patient will be considered a good adherent when he answers properly the complete questionnaire.

The dose/pill count is the number of pills or doses taken divided by the number of pills or doses prescribed, multiplied by 100 (expressed as a percentage). According with Sackett et al. [21] recommendations, a good adherence is considered when the result of counting is between 80% (a twenty percent of doses/pills missed) and 110% (the patient inhales a ten percent more of doses/pills) of dose/pill prescribed. This cutoff point was selected for consistency with other studies [24-26].

We consider the dose/pill count as the more confident method. We use the two self-reported methods because they are easier to apply in clinical practice and, in a previous work conducted by us, we found acceptable indicators of their validity as diagnostic test for treatment adherence in COPD patients under certain environment circumstances.

#### Secondary outcome

functional status (spirometry), quality of life (St George respiratory questionnaire [27], and EuroQoL-5 D [28]) and the clinical progress with SeguiEPOC questionnaire.

#### Independent variables

age, sex, educational level, comorbidity, smoking history, COPD severity grade (according to SEPAR guidelines [29]), prescribed medication, family support (Family Apgar Test [30]): social support (DUKE-UNC Test [31]).

### Intervention

#### Activities

The intervention has two main activities: a group session followed by individual visits.

##### Group session

the session will last 2 hours including the patient's reception, the material distribution, the intervention and the farewell. The intervention is structured in three parts: the first part will be conducted following the focus group methodology. The number of patients will be about 6 to 9 per group. In the second part, patients will receive information about their disease and treatment with the aid of the distributed material. The third part will be conducted as a training session about inhalation technique.

##### Individual visits

will last 30 minutes approximately, it will depend on the topics related to adherence that we will need to work with every patient. These encounters will be videotaped to facilitate the collection of the data. In addition to written informed consent including in the recruitment visit, we will ask for their verbal informed consent all the times that we will videotape a visit.

#### Intervention Content

The content of the intervention can be divided in three elements that include the most relevant aspects related to the non-adherence in patients with COPD: motivational and cognitive aspects, and skill development.

##### Motivational Aspects

We mean to find out why the recommended therapeutic regime is not accomplished, in order to use the individual motivational aspects that could improve the adherence over the next follow up visits.

This aspect of the intervention will be developed along the first part of the group session and in the individual visits with different methodologies. In the first part of the group session, we will use qualitative methods by the *focus group technique *to explore the beliefs and attitudes of patients. In the individual visit, we will use this knowledge to design individual interviews to work on motivational aspects to try to improve treatment adherence. These individual interviews will be conducted in the follow-up visits (V1, V2 and V3).

Qualitative research methods have proved to be useful in the health care field. These methods provide in-depth knowledge about perceptions, beliefs and values of the persons or groups involved [32]. Taking into account the importance to explore behaviours and attitudes of our target population we consider the group interview (*focus group*) will be the most adequate technique to gather the information. These techniques explore dynamics interactions between group members, consider issues that may underlie individual preferences and explore areas of agreement and dissent [33], to reveal patient's experiences and points of view about their illness and treatment.

**Figure 2:**
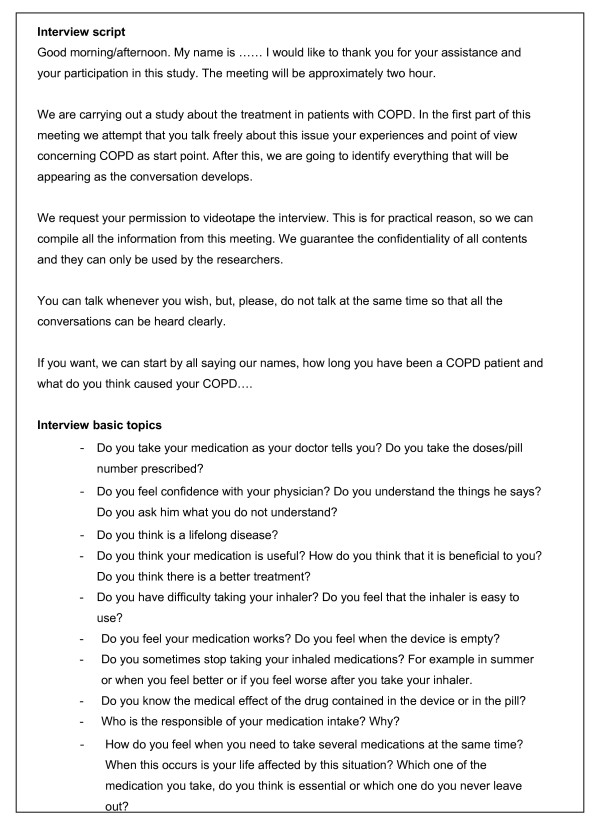
**Focus group interview script**.

The research team has determined the basic topics to explore in the focus group. These are about adherence and the strategies to cope with the disease, and they are made as a semi-structured questionnaire (Figure 2). The content of this questionnaire might be adapted depending on the findings that could arise during the development of the focus group [33].

An interviewer and a collaborator will be present in each group. They are both specially trained in qualitative techniques and in the use of inhaler devices. The training in motivational techniques has been done under one of the project researcher's supervision who is an expert in communication techniques at the Teaching Unit of Community and Family Medicine (Málaga).

The interviewer will introduce the study's objective, will explain the need to video-record the session and will give information about data confidentiality. The patients are asked for their consent to videotape the session. After the group sessions the two investigators will discuss their impressions and notes about each group.

The data will be extracted using a form designed by the research team using the group session questionnaire. All the participants' opinions will be collected. Each group will be reviewed and the data will be collected from the videotape by at least two members of the research team. After the data collection, these researchers will discuss their opinions of each patient and will agree a motivational strategy for the individual visits.

In the individual interviews, we will work with the patient on all aspects that we have detected in the focus group using the techniques of the motivational interview. These visits will be video-recorded and the data will be collected in the same way as in the focus group to design the strategy for the next follow-up visit.

##### Cognitive Aspects

Information is given about the disease so the patients can get to know it better and be more conscious about the importance of the scheduled treatment.

We have designed an audiovisual presentation to inform the patients about the most important issues of their illness and its treatment and a written dossier with the oral presentation content which will be given to the patient.

In the follow-up visit we will ask the patient if he has doubts about his illness or about the information he was given in the group session. Any questions they have are answered straightaway.

##### Skills Training

In the group session, the patients will receive inhalation technique training, about how they use their inhalers, why a good technique is important and will practice the proper technique according to SEPAR guidelines [34]. The training in the use of inhaler devices has been done at the Pediatrics Pneumology Service of Hospital Materno Infantil (Málaga).

We will perform training in three steps:

- Patients will be asked how they use their inhalers. Using a variety of placebo inhalers, all of them will demonstrate how they use them to the instructor.

- When all patients have done the demonstration, the trainer will ask about the problems and mistakes with each technique. At the beginning, these questions will be for individuals, but when all patients have answered individually, the questions will be made general and opinions can be expressed about any demonstration.

- The trainer will demonstrate the proper technique. Each device will be used and its technique will be explained step by step. The importance of following the correct technique every time the patient uses the inhaler device will be emphasized.

Finally the patients can ask questions and they will practice the techniques until they are correct or until the patient becomes tired.

In the follow-up visits we will review the inhalation technique and we will solve any mistake or doubt as explained previously. The objective here is for the patients to identify their mistakes, and if they cannot, to remind them the proper technique by as many demonstrations as are necessary.

### Recruitment

Patients will be contacted using their health center records. They will be invited to participate in the study after a brief explanation by telephone about the research aims and they will receive an appointment in the health center. At this first appointment (inclusion visit) patients will receive more detailed information about the study and if they agree to participate, they will sign the written consent.

At this point, subjects will be divided randomly into two groups using the block randomization technique. The blocks consist of 4 patients, two subjects per group [Intervention (I) and control (C)]: (IICC); (CCII); (CICI); (ICIC); (ICCI); (CIIC). Consequently, the numbers in the two groups never differ at any time by more than two. The blocks will be marked with a number from 1 to 6 and we will choose blocks at random to create the allocation sequence using a sequence of random numbers generated by Microsoft Excel 2003 program with the function fx:RAND(). We will need 35 random blocks; and the final list of the included patients in each arm of the trial will be set in advance. The researcher who will allocate the patient will have the open-list of randomization, but there will be no change of patient group related with the subjects characteristics. Because the patients are from 6 health centers, the randomization and the presence of the intervention and control subjects in all the health centers will be guaranteed.

After randomization all the study data will be recorded and the adherence will be measured in both groups.

### Follow up

#### Control Group

they will be appointed for:

Visit 1: It will take place 3 months after the inclusion. It will involve the measure of adherence and other variable changes.

Visit 2: It will take place 6 months after the inclusion. It will involve the measure of adherence and other variable changes.

Visit 3: It will take place after 1 year follow up. All the study data will be recorded including treatment adherence.

There will be a pre-adherence visit before each follow-up visit except for the inclusion one. The reason for the pre-adherence visit is to ask the patient to bring a new container that will be marked with the date in which it is opened in order to count doses/pills through the month. This is done because most of the devices that patients use last 30 days. The pre-adherence visit will be done by telephone to guarantee the follow-up.

#### Intervention Group

After the inclusion, the first part of the intervention (group session) will be carried out in each health center and the intervention group will receive appointments for:

Visit 1: It will take place 3 months after the intervention. It will involve the measure of adherence and other variable changes, encouragement about inhalation techniques and motivational aspects related to treatment adherence.

Visit 2: It will take place 6 months after the intervention. It will involve the measure of adherence, other variable changes and encouragement about inhalation techniques and motivational aspects related to treatment adherence.

Visit 3: It will take place after 1 year follow-up. All the study data will be recorded and encouragement about inhalation techniques and motivational aspects related to treatment adherence.

There was a pre-adherence visit prior to all visits as explained previously.

Adherence will be measured at each visit to control its evolution during the follow-up period. All individual follow-up visits will be videotaped to identify the motivational aspects and skills which each patient needs to be applied at the next visit.

### Statistical Analysis

A descriptive statistical analysis will be performed for all the study variables. We will calculate the mean, median and standard deviations for quantitative variables, and the absolute and relative frequency for qualitative variables.

The 95% confidence level will be applied. The analysis will be made under an intention-to-treat procedure. The between-group comparison for the primary outcome will be explored using the Chi Square Test. The Relative Risk Reduction (RRR), the Absolute Risk Reduction (ARR) and the Number Needed to treat (NNT) will be calculated. Inferences for the secondary outcomes will be made using an analysis of variance (ANOVA). Finally a logistic regression model will be performed for the primary outcome [treatment adherence (yes/not)], considering the intervention as the predictive variable and the rest of the independent measures as the possible modifying factors. We will use the usual 5% significance level (α = 0.05) and the SPSS statistical package, version 15.0, to run the proposed analysis.

### Study limitations

The main study limitation is the selection bias due to the missing data. In order to diminish this bias, we will apply several strategies:

- An increase of 15% in the sample size (expected losses)

- Three phone calls at different days and times for unreachable patients

- Rescue appointments for the non-assistants to visits (three different appointments)

Other aspect is the Hawthorne effect along the study (ie, tendency of subjects participating in a research study to change their behaviour). Although this could affect overall estimates the adherence, the implications might be less important in comparing results of different measurements tools (unless, of course, the effect is differentially captured by each measurement tool); furthermore, it is difficult to perceive that any potential Hawthorne effect would be maintained over the many months of study.

In addition we take into account the possible contamination between the control and the intervention group because of the relationship between subjects in their daily life (neighborhood, relatives, social networks or associations). However, in another educational intervention study performed with obese patients in our area we did not find a significant level of this effect [35]. We also believe that the intervention characteristics (several steps, small groups and individual visits) that we have described will have little influence on contamination between groups.

Another important aspect to consider is the protocol and the intervention standardization. This is why the dynamic has been structured in an exhaustive way and the intervention will be performed by two professionals trained in communication, disease knowledge and inhalation techniques of the different devices used by COPD patients. Furthermore, we have designed a manual for the researchers where we explain the working plan, the different parts of intervention (the group session and the individual visits), the protocol scheme to know what they have to measure each time and the details to asses each variable included in the study. In this way, the procedure can be replicated elsewhere.

## Discussion

We have described the treatment adherence in a group of patients in the Málaga area in the previous studies and we have tried to validate easier and faster methods than the dose/pill count, to evaluate the non-adherence magnitude in these patients. From the results of this previous work we have learnt to measure better the treatment adherence. Once we have described the problem, the next step is to design a strategy to improve it. There is little evidence about the use of strategies to improve adherence in COPD patients. However the non-adherence magnitude in these patients is high and it affects directly their objective and subjective health outcomes. Therefore we believe that the design and development of an intervention to improve adherence in COPD patients is very important to reach this purpose; we also need to analyze deeply the motives and barriers or difficulties that these patients have to comply with the recommended medication regimens.

Adherence to the prescribed treatment involves a behavioral change. Cognitive, emotional and motivational aspects have an influence on this change so this is the reason why we consider that the best intervention procedure to improve adherence would be a strategy that takes into account cognitive and emotional aspects of patients and that could be applied in the daily clinical practice.

Our hypothesis is that the application of a multifactor intervention to COPD patients under scheduled inhaled treatment will increase up to 25% the percentage of adherent patients in this group in comparison to the control group. This intervention could be feasible to implement in the clinical practice context. With this strategy we try to promote patients' autonomy and responsibility about their disease, achieving in this way a greater improvement on their health outcomes and increasing clinical effectiveness in our daily practice.

## Competing interests

The authors declare that they have no competing interests.

## Authors' contributions

1) PBF has been involved in drafting the manuscript and writing it. She has participated in the design of the study and the intervention.

2) JLF has been involved in the design of the study and he has participated in reviewing the manuscript.

3) FVE has been involved in the design of the intervention and she has participated in reviewing the manuscript.

4) AGR has been involved in the design of the study and he has participated in reviewing the manuscript.

5) DPT has been involved in the design of the study, and he has participated in reviewing the manuscript.

6) FLF has been involved in drafting the manuscript and writing it. She has participated in the design of the study and the intervention.

All authors read and approved the final manuscripts.
